# Association between Levels of Loneliness, Laboratory Measurements, and Behavioral Aspects in a Primary Care Setting in Crete, Greece

**DOI:** 10.3390/ejihpe14040069

**Published:** 2024-04-18

**Authors:** Panagiotis Volkos, Manolis Linardakis, Panagiotis Stachteas, Foteini Anastasiou, Athina Tatsioni, Marilena Kampa, Emmanouil K. Symvoulakis

**Affiliations:** 1 Fourth Local Health Team—Academic Unit of Heraklion, 71303 Heraklion, Greece; 2 Department of Social Medicine, School of Medicine, University of Crete, 70013 Heraklion, Greece; 3 Laboratory of Primary Health Care, General Practice and Health Services Research, School of Medicine, Aristotle University of Thessaloniki, 54124 Thessaloniki, Greece; 4 Fourth Local Primary Care Team (TOMY), Municipality Practice, Academic Practice of Heraklion, University of Crete, 71303 Heraklion, Greece; 5 Research Unit for General Medicine and Primary Health Care, University of Ioannina, 45110 Ioannina, Greece; 6 Laboratory of Experimental Endocrinology, School of Medicine, University of Crete, 70013 Heraklion, Greece; 7 Clinic of Social and Family Medicine, Faculty of Medicine, University of Crete, 70013 Heraklion, Greece

**Keywords:** atherosclerotic index, behavior, loneliness, social contacts, triglycerides

## Abstract

This paper examines potential associations of loneliness with laboratory data and specific psychosocial and behavioral attitudes. The sample collection took place in an urban Primary Health Care unit between May and July 2023, consecutively, and once exclusion criteria were implemented. Participants were aged between 40 and 75 years. Routine laboratory test results upon study initiation and six months before were used. The University of California, Los Angeles (UCLA), Loneliness Scale (Version 3), blood glucose, serum lipids, Fibrosis-4 index, and Creatinine Clearance (CrCl) were assessed through hierarchical multiple logistic regression analysis. Based on full model (3rd) analysis, those who were engaged in an individual sport or activity or had contacts with more friends presented significantly lower odds for increased loneliness levels (odds ratio (OR): 0.28 [95% confidence interval (CI) 0.09–0.91], *p* = 0.034 and OR: 0.76 [95%CI 0.66–0.88], *p* < 0.001, respectively). The consumption of alcohol was associated with increased loneliness (OR: 5.55 [95%CI 1.42–21.63], *p* = 0.014). Elevated triglyceride levels were linked with moderate or no loneliness (OR: 0.20 [95%CI 0.05–0.83], *p* = 0.026), while an increased LDL/HDL atherosclerotic index was related to increased subjective loneliness (OR: 4.50 [95%CI 1.12–18.13], *p* = 0.035). The need for holistic approaches—involving primary care personnel—in understanding and addressing loneliness, recognizing its multifaceted nature as well as the diverse factors that contribute to this issue, is considered challenging.

## 1. Introduction

Loneliness is a common feeling; it remains unpleasant and undesirable and many people try to buffer it [1]. As Victor and Yang (2012) reported, loneliness increases among young adults (under the age of 25 years), decreases during middle age, and deteriorates again among older adults aged 65 years and over. Furthermore, it was reported that the “quantity” of social relationships plays an important role in the younger ones, while “quality” is more essential in the elderly [2].

In general, loneliness is related to several health problems, including cardiovascular diseases. The presence of loneliness or social isolation leads to a risk increase of coronary heart disease by 29%, while social isolation is also associated with a higher risk of stroke by 32% [3]. Moreover, individuals who report feeling lonely were found to have higher odds of an increased cholesterol level (1.31 times), the presence of diabetes (1.40 times), and depression (2.78 times) [4]. Additionally, augmented loneliness and limited social participation were linked with a greater risk for dementia, according to the meta-analysis of Kuiper et al. (2015) [5]. From the aforementioned studies, it could be argued that loneliness interplays, in various ways, with health and disease. Therefore, by linking this feeling with laboratory markers, some valuable insights about a neglected health risk might be delivered.

Given that depression and loneliness were previously linked [4], mental health issues should be taken into consideration when studying the impact of loneliness on physical well-being. More specifically, it was reported that higher levels of loneliness are associated with increased odds of metabolic syndrome, although, according to the authors, that relationship was mediated by the occurrence of depression [6]. In addition, loneliness was found to be correlated with a greater risk of metabolic syndrome, a higher level of triglycerides, and lower levels of high-density lipoprotein cholesterol (HDL-C) for people with psychotic disorders [7]. According to the above, it appears that mental health issues are, somehow, related to loneliness and several physical conditions, without having specific knowledge about the exact operating mechanism.

In the literature, loneliness was studied alongside social inclusion (or exclusion) and social isolation, and given the fact that these social terms are content and effect-connected, they may be positively or negatively predictive for loneliness. According to the study of Dahlberg et al. (2022), social exclusion indicators such as participation in the community, social relationships, material resources, and the neighborhood are associated with an increased likelihood of loneliness in Nordic countries [8]. Other than that, social exclusion may cause feelings of lower self-esteem, anger, depression, anxiety, and self-destructive behavior [9]. The effects of social inclusion and isolation on physical health have been investigated, showing that higher levels of social inclusion are linked with lower blood glucose and low-density lipoprotein (LDL) [10]. Greater social isolation was correlated with two-fold increased odds of deterioration in renal function and the presence of chronic kidney disease [11]. According to the review of Holt-Lunstad (2022), it is concluded that social connections should be addressed as a public health issue [12].

All of the above are showing that loneliness is a feeling that can influence health to a great extent and needs to be taken into account for both prevention and treatment purposes, especially when considering that several societies tend to become more individualistic than collective [13]. Relating laboratory data to an easy-in-use tool for loneliness may provide critical information to a health professional team, in order to take preventive action at a psychosocial, behavioral, and physical level.

The primary objective of this study is to examine potential associations of loneliness with laboratory findings within a primary care setting. The secondary aim was to triangulate scale scores—detecting loneliness—with routine laboratory results and other meanings of psychosocial and behavioral attitudes.

## 2. Materials and Methods

This study followed an observational, cross-sectional design and took place in the 4th TOMY (Topiki Monada Ygeias—Local Health Unit) of Heraklion, Crete, Greece, which is an urban Primary Health Care (PHC) unit. The study population targeted all of the registered adults of the unit between 40 and 75 years of age. The participants had to be able to read, write, and comprehend the Greek language, with Body Mass Index (BMI) ≤ 29.9 kg/m^2^, without major head trauma or major psychiatric disorder, and those who were not pregnant or breastfeeding during the study period. The sample was collected between May and July 2023 and 120 participants were included, from 288 scheduled appointments during two morning sessions weekly, in a consecutive manner, after exclusion criteria were applied. Routine laboratory test results upon study initiation and six months before were used. Patients were asked to provide further information whenever needed. STROBE checklist for cross-sectional studies was strictly followed.

### 2.1. Data Collection

A sheet to collect information was designed for the purposes of the present study. It contained information regarding age, gender, marital status, level of education, occupation, height, weight, smoking habits, consumption of alcohol, chronic diseases, prescribed medications, existence of any psychiatric diagnosis, the number of friends they came into contact with, either face-to-face or via electronic means, during the last six months, level of individual physical activity or sport during the last year, and experience of a dramatic event in the family during the last year. For the purposes of this study, blood glucose (mg/dL), total cholesterol (mg/dL), triglycerides (TG) (mg/dL), HDL-C (mg/dL), LDL-C (mg/dL), aspartate aminotransferase (AST) (U/L), alanine aminotransferase (ALT) (U/L), platelet count, and creatinine (mg/dL) were tabulated. Also, Fibrosis-4 (Fib-4) score, modified Creatinine Clearance (CrCl) (mL/min/1.73 m^2^), and HDL-C/LDL-C index were calculated. 

### 2.2. University of California, Los Angeles (UCLA), Loneliness Scale (Version 3)

The feeling of loneliness was measured using UCLA Loneliness Scale, Version 3 [14]. The scale assesses feelings of perceived loneliness and comprises 20 items (11 with negative and 9 with positive meanings). Answers are based on a 4-point Likert-type scale, with never = 1, rarely = 2, sometimes = 3, and always = 4. To measure the outcome of the scale, 9 items need to be reversed (items 1, 5, 6, 9, 10, 15, 16, 19, and 20) and then all of the items need to be summed up. The final score range is 20–80. Higher score indicates greater feelings of subjective loneliness. The cut-offs used were those reported by Lee and colleagues (2021): total score < 28 as absence or low sense of loneliness, 28 to 43 as moderate feeling, and >43 as high feeling of loneliness [15,16]. The scale was validated in Greek [17]. Cronbach α was estimated at 0.880.

### 2.3. Cut-Offs for Laboratory Variables

The following cut-offs were used to separate normal and abnormal findings: in total cholesterol > 200 mg/dL or receiving medication, in TG > 150 mg/dL or receiving medication, in LDL > 160 mg/dL or receiving medication, in HDL <40 and <50 mg/dL for men and women, respectively, in blood glucose 100+ mg/dL or receiving medication, in Fib-4 1.45+ [18], and in CrCl < 90 mL/min/1.73 m^2^ [19,20]. For the atherosclerotic index LDL-C/HDL-C, the cut-off was 2.517 [21]. 

### 2.4. Ethics

The approval to conduct the study, for the needs of an ongoing PhD thesis, was obtained from the Ethics and Deontology Committee of the University of Crete (protocol number: 166/11.11.2022). The research protocol was additionally approved by the 7th Health Regional Authority of Crete (protocol number: 6460) and the study was performed in accordance with the Helsinki Declaration. Written informed consent was obtained by the participants.

### 2.5. Statistical Analysis

Data analysis was implemented using the SPSS program (IBM Corp. Released 2019, IBM SPSS Statistics for Windows, v.25.0, Armonk, NY, USA: IBM Corp.). Frequency of absolute and relative distributions and measures of location and dispersion of descriptive and laboratory features of the participants were estimated. Score levels of UCLA scale, blood glucose, serum lipids, Fibrosis-4 index, and CrCl were also estimated, as well as the frequencies of their higher levels. Hierarchical multiple logistic regression analysis was implemented in higher levels of loneliness and in relation to basic characteristics, health habits, blood glucose, serum lipids, Fibrosis-4 index, and CrCl of 120 patients–attendees. Acceptable level of significance was set at 0.05.

## 3. Results

The mean age of participants was 59.8 (standard deviation (SD) = 9.5) years, and most were women (73.3%, *n* = 88). Table 1 presents the baseline characteristics of the 120 patients–attendees within the PHC setting of the current study. Regarding the educational level, 25% (*n* = 30) of the participants reported tertiary education, whereas MSc or PhD degrees were found to be held in 2.5% (*n* = 3) of the patients.

Regarding the behavioral risk factors of the participants, current smokers represented the 30% (*n* = 36), while the alcohol consumption of one or more drinks per week was equally prevalent (30%, *n* = 36). The mean BMI was 26 (SD = 2.9) kg/m^2^, and remarkably, only 34.2% (*n* = 41) of patients–attendees had a BMI bellow 25 kg/m^2^. With regards to chronic medical conditions, only 13.3% (*n* = 16) of the participants reported having no chronic disease, whereas the prevalence of mental health disorders was accounted for by 37.5% (*n* = 45). Nevertheless, hyperlipidemia (30%, *n* = 36) was the most prevalent physical medical condition followed by diabetes mellitus (28.3%, *n* = 34) (Table 2).

Among the 120 participants, the perceived feeling of loneliness was found with a mean score of 41 (SD = 10.1), as assessed with the UCLA Loneliness Scale, Version 3. More particularly, 109 (90.8%) participants reported moderate or high levels of subjective loneliness, while only 11 (9.2%) participants reported the absence or low feeling of loneliness (Table 3). Laboratory tests revealed impaired renal function and decreased levels of creatinine clearance (mean = 77.4, SD = 21.8) and a good lipid profile with normal levels of total cholesterol (mean = 188.5 mg/dL, SD = 37.9), HDL-C (mean = 59.5 mg/dL, SD = 18.4), LDL-C (mean = 108 mg/dL, SD = 29.9), and triglycerides (mean = 110.3 mg/dL, SD = 58.6), and a mildly impaired glucose metabolism (mean = 104.4 mg/dL, SD = 25.3).

Table 4 presents the association of the increased levels of loneliness assessed by the UCLA Loneliness Scale with participants’ demographic and laboratory characteristics after using hierarchical multiple logistic regression. Initially, according to the first model, married people seemed to have significantly lower odds for an increased sense of loneliness (odds ratio (OR): 0.39 [95% confidence interval (CI) 0.17–0.91] *p* = 0.030). In the second model, personal characteristics were not significantly related to the feeling of loneliness, while participants who were engaged in an individual activity or sport during the last year seemed to have significantly lower odds for an increased feeling of loneliness (OR: 0.29 [95%CI 0.10–0.84], *p* = 0.023). Moreover, the number of social contacts (each additional friend with whom any participant routinely came into contact with (either face-to-face or via electronic means) was strongly related to significantly lower odds for feeling lonely (OR: 0.82 [95%CI 0.73–0.93], *p* = 0.001). Likewise, in the third model of hierarchical multiple logistic regression, where the laboratory results (glucose, blood serum lipids, fibrosis index, and creatinine clearance) were incorporated, participants with elevated triglyceride levels were associated with moderate or no loneliness (OR: 0.20 [95%CI 0.05–0.83], *p* = 0.026), while those with an increased LDL/HDL atherosclerotic index had an increased subjective feeling of loneliness (OR: 4.50 [95%CI 1.12–18.13], *p* = 0.035). Interestingly, participants who were engaged in an individual sport or activity or had more social contacts with friends presented significantly lower odds for an increased feeling of loneliness (OR: 0.28 [95%CI 0.09–0.91], *p* = 0.034 and OR: 0.76 [95%CI 0.66–0.88], *p* < 0.001, respectively). Finally, the consumption of alcoholic beverages within the week was associated with an increased feeling of loneliness (OR: 5.55 [95%CI 1.42–21.63], *p* = 0.014).

## 4. Discussion

The present study focused on assessing the levels of loneliness in primary care attendees and exploring potential associations with demographic, behavioral, and routine laboratory test results. Among participants, mental health disorders were prevalent in 37.5% of participants and the most frequent physical medical conditions were hyperlipidemia (30%) and diabetes mellitus (28.3%), while laboratory tests overall showed an impaired renal function, acceptable lipid profile, and mildly impaired glucose metabolism. The perceived feeling of loneliness was moderately reported, while nine out of ten participants reported moderate or high levels of subjective loneliness. The association of loneliness with demographic and behavioral characteristics showed that married individuals had lower odds of feeling lonely; however, this association diminished in the subsequent models of analysis. Engagement in individual activities or sports, a higher number of social contacts reported, and a lack of weekly alcohol consumption were found to be related with decreased loneliness. Regarding the laboratory tests, elevated triglyceride levels were associated with moderate or no loneliness, while an increased LDL/HDL atherosclerotic index was linked to increased levels of loneliness. 

The finding that a consistent proportion of primary care attendees report moderate or high levels of loneliness aligns with the existing literature, indicating that loneliness is becoming a growing community health issue and a prevalent subject for discussion in healthcare settings [22]. Loneliness and social isolation are both linked to negative health consequences, such as a diminished health status, poor quality of life, increased utilization of healthcare services, and increased morbidity and mortality [23,24,25]. According to a cross-sectional study conducted in Colorado, USA, the prevalence of loneliness was estimated to be about 20% among adults visiting outpatient primary care facilities [26]. Nevertheless, especially among the elderly, social isolation and loneliness are so frequent that they are currently considered to be the new geriatric ‘giants’ [27]. However, according to a multi-state study, one third of respondents under the age of 25 reported that they had experienced loneliness, in contrast to one out of ten within the group aged over 65 years [26]. Therefore, attention to detecting signs of loneliness should be paid regardless of a person’s age.

Furthermore, the initial observation that married participants had lower odds of feeling lonely is consistent with some previous studies, suggesting that their marital status can influence feelings of loneliness and perceived social support [28]. In a sample with older individuals, divorced and widowed persons described higher levels of loneliness, in comparison with the married ones [29]. Also, gender should be taken into account, since the utilization of outpatient health care was positively correlated with loneliness in women but not in men [30]. Additionally, the protective role of engagement in individual activities or sports and the reported social contacts are consistent with previous research, emphasizing the positive impact of social engagement and connections on mental well-being in general [31,32,33]. Both the present study’s results and the existing literature underline the importance of the aforementioned behavioral factors that may play a protective role against the adverse effects of loneliness. Furthermore, previous studies have shown that, at an individual level, social interaction, social support, and behavioral interventions which concentrate on enhancing social skills and addressing maladaptive social cognition have been noted to effectively alleviate loneliness [25,34], while in seniors, taking care of their grandchildren or undertaking volunteering activities may show similar results [35]. This suggests that promoting and facilitating such initiatives may have positive implications for mitigating loneliness.

According to our findings, the consumption of alcoholic beverages weekly was associated with a fivefold increased feeling of loneliness, although the association between alcohol consumption and loneliness is not well established in terms of cause or consequence. It is known that experiencing loneliness, conversely approached, has the potential to compromise self-regulatory abilities, leading to an increased susceptibility to noxious behaviors such as excessive alcohol consumption [36]. However, within a cohort of community-based adults aged 50 and above, loneliness was associated with a decreased frequency of alcohol consumption [37]. When studying loneliness and health behaviors, micro- or macro-circumstances, at a personal, cultural, or social level, can influence an already complex phenomenon, whereas the demand for variables or methodologies, toward this purpose, remains challenging [38]. Therefore, additional research is needed in order to understand how loneliness and alcohol are related and identify the possible mediating factors, taking into consideration gender, ethnicity, culture, social status, and personal motivations.

Incorporating routine laboratory data into the analysis provided novel insights into the comprehensive assessment of the relationship between physical health and loneliness. This is particularly noteworthy as a limited body of the literature has delved into the direct correlation between specific laboratory parameters and the subjective experience of loneliness. Elevated triglyceride levels [6,7,39] and diminished HDL-C levels [6,7] have been previously observed in individuals experiencing loneliness. However, there was no discernible correlation between total cholesterol, LDL-C, and non-HDL-C levels with loneliness [40]. Notably, in our study, participants with elevated triglyceride levels were associated with lower odds of feeling lonely. It could be hypothesized that people who feel less lonely may make diet choices as part of a more active social life. In our sample, obese participants were not included and this design peculiarity may be related to our finding about elevated triglycerides presented. However, further investigation is needed to establish solid conclusions. On the contrary, an increased LDL/HDL atherosclerotic index was linked to increased loneliness. This finding is important since the overall lipid profile within study participants was initially assessed as good. The aforementioned information suggests a potential interplay between the lipid metabolism and subjective feelings of loneliness, indicating a need for the further exploration and understanding of the latent mechanisms. Future research could focus on mental health issues and how they are related to physical and lifestyle factors.

Apart from the connection between loneliness and laboratory results, several studies have linked loneliness directly to chronic conditions. For example, both greater loneliness [41] and impaired social health (which includes loneliness) [42] have been associated with cardiovascular disease. Additionally, individuals reporting the occurrence of chronic obstructive pulmonary disease demonstrated a higher incidence of social isolation and loneliness in comparison with the rest of the participants [43]. There is also some evidence showing an association between loneliness and dental health or even tooth loss [44]. Dental health is a neglected issue that deserves more attention as a public health priority and it is noteworthy to mention such an association for many reasons. Furthermore, loneliness may have a pluripotent burden on human lives since, apart from its impact on physical well-being, it was reported to be related with mental health issues, as mentioned earlier. More specifically, loneliness was linked with depression [45,46], anxiety [47], eating disorders [48], or even mortality caused by suicide [49]. By ‘Puzzling’ the mentioned ‘pieces of knowledge’, it can be understood that loneliness appears to influence many dimensions of physical and mental health with a direct manner, without being able to precisely assess its multiple impact on life, when more than one dimension are synchronically affected overtime.

Understanding the factors associated with loneliness within primary care attendees has significant implications for healthcare interventions. Tailoring interventions to address not only behavioral aspects, but also considering laboratory data may enhance knowledge integration. Strategies that promote social engagement and support networks may be proved valuable in mitigating feelings of loneliness [25,34]. The healthcare system constitutes a pivotal yet underutilized partner in endeavors aimed at discerning, averting, and alleviating the negative consequences of social isolation and loneliness [50,51]. Concerning primary care, a better quality of the offered services may lead to decreased loneliness scores among attendees [52]. Therefore, family physicians and other healthcare providers, simultaneously with screening for conventional risk factors (like smoking, physical inactivity, alcohol consumption, and diet), should systematically screen to detect individuals who may be experiencing isolation, loneliness, or social vulnerability [27]. Subsequently, they should suggest evidence-based and patient-centered interventions aiming at fortifying social connections for such patients [24]. In fact, there is evidence that group interventions implemented by primary care professionals have shown promising results in addressing loneliness [53]. Although with another, and reversed, meaning, primary care and loneliness deal with health and social dimensions of life, in terms of magnitude and mutual interference. For this reason, developing a primary care ability to detect and manage loneliness goes beyond a conventional debate. However, additional research is imperative to furnish conclusive guidance regarding the specific effectiveness of interventions designed for distinct populations and underlying mechanisms [24,27,54], or to determine whether social resources are useful in alleviating loneliness. For example, surveys could focus on the design and intergenerational implementation of educational programs for both older adults [55] and adolescents and young adults [56]. These initiatives might be tailored and adopted in the context of the community to gain loneliness coping skills as individuals.

### Strengths and Limitations

The combination of laboratory measurements and the identification of novel associations bring a fresh perspective to the understanding of loneliness within primary care attendees, at least in terms of the medical perception. While this study provides some valuable insights, several limitations should be acknowledged. This study sample is relatively small and may not be representative of broader population groups, so findings should be discussed with caution. Small or moderate samples may lead to an overestimation of the effect measure, although this phenomenon also depends on the data structure [57]. However, in the present study, structure issues did not appear. This overestimation, in samples above 100, might be tolerable as it does not appear to have any relevance for the interpretation of the results in the context of a single study since it is much lower than the standard error of the estimate, and this eventuality should be assessed when small studies are pooled together [57].

Nevertheless, the present survey did not intend to project these finding with any generalization for the entire primary care population. On the other hand, the application of the criteria, such as a certain range of age (40–75) and BMI (≤29.9/m^2^), led to obtaining less uncertainty with the emerged associations. For example, it is not well established how Millenials perceive loneliness [58] or how obesity interacts with loneliness and social isolation [59]. In this way, the detected levels of loneliness do not appear to be overestimated and all the associations which have emerged show a socio-behavioral linkage (alcohol consumption, sport activity, routine contacts, and lipid profile).

In addition, the cross-sectional nature of the study restricts the founding of causation, while the self-reported nature of the information on behavioral aspects may introduce bias. Additionally, a different source, time, and process occurring within the laboratory tests included, from a period of six months, may lead to a further limitation. However, in our opinion, this asynchronous integration of information, for research meanings, that bridge social with clinical inputs, may be interesting since many of the variables socially or behaviorally investigated might have a retrospectively longitudinal projection with loneliness. 

Furthermore, it is imperative to note that loneliness could be correlated with the diminished utilization of healthcare services and attenuated solicitation for medical interventions. Therefore, it is conceivable that our study may not accurately capture the exact prevalence of loneliness at a unit coverage level. Finally, the prevalence of mental and physical health conditions may differ from existing epidemiological or local community data. Future research should consider longitudinal designs and provide useful insights into the dynamic nature of loneliness and its associations with socio-demographic and behavioral characteristics and laboratory markers over time, incorporating a broader and more representative sample from multiple primary care settings. This can contribute to extrapolating more solid observations regarding loneliness, moving towards the interface between health and disease.

The present study did not include individuals with obesity or morbid obesity. In an interesting systematic review, focusing on obesity and loneliness or social isolation, while some studies point to an association between increased loneliness and obesity, the authors recognized that mixed observations occur [59]. Moreover, the rationale of including participants between 40 and 75 years of age was based on previous findings from primary care settings in Crete that used a similar population group—above 40 years of age—investigating local trends of cardio-metabolic morbidity [60]. Also, a different impact of loneliness may occur among younger adult generations, as has been shown in a study that included different age groups during the early COVID-19 pandemic period [58].

## 5. Conclusions

In conclusion, involvement in individual activities or sports, higher numbers of social contacts reported, and a lack of weekly alcohol consumption and elevated triglyceride levels were associated with moderate or no loneliness, while an increased LDL/HDL atherosclerotic index was linked to increased levels of loneliness. This preliminary analysis offers some new information on socio-demographic, behavioral, and routine laboratory test results and how these variables interplay with perceived loneliness among primary care attendees. This study’s findings underscore the need for open-minded approaches and variable selection when addressing meanings of loneliness, recognizing its pluripotent effect, as well as the different aspects that may contribute to its pervasive complexity. Primary care may offer promising attributes in terms of research findings that emerge from a mixed methodology and socio-iatrogenic thinking. Further research aiming at understanding similar associations may discuss tailored interventions for loneliness and neglected mental and physical circumstances or discussions within primary care settings.

## Figures and Tables

**Table 1:** Basic characteristics of 120 patients–attendees within primary care setting of current study.

		*n*	%
Gender	male/female	32/88	26.7/73.3
Age, years	mean ± stand. dev.(min, max)	59.8 ± 9.5 [40, 75]	
Family status	unmarried, divorced, widow	39	32.5
	married	81	67.5
Children	none	17	14.2
	1	11	9.2
	2+	92	76.6
Education level	Primary school	24	20.0
	Junior high school	13	10.8
	High school	34	28.3
	Technical education	19	15.9
	University/Technological School	27	22.5
	MSc, PhD	3	2.5
Occupation	employed	51	42.5
	unemployed, retired	69	57.5

**Table 2:** Health habits and characteristics of 120 patients–attendees within primary care setting of current study.

		*n*	%
Body Mass Index, kg/m^2^	mean ± stand. dev.(min, max)	26.0 ± 2.9 (18.6, 29.8)	
	normal	41	34.2
	overweight	79	65.8
Smoking	yes	36	30.0
Alcohol consumption, drinks per week	none	84	70.0
	1+	36	30.0
Conditions, chronic diseases ^a^	yes	104	86.7
	mental disorder	45	37.5
	hyperlipidemia	36	30.0
	diabetes mellitus	34	28.3
	hypothyroidism	21	17.5
Routine contacts or meetings with friends during the last 6 months	mean number (median) [min, max]	8 (6) [0, 25]	
Personal sport/activity during the last year	yes	49	40.8
Recent dramatic event in family during the last year	yes	44	36.7

^a^ Some participants displayed co-morbidity.

**Table 3:** Levels of University of California Los Angeles Loneliness Scale (UCLA), blood glucose, serum lipids, Fibrosis-4 index, and Creatinine Clearance (CrCl) of 120 patients–attendees within primary care setting of current study.

	Mean	Stand. Dev.	Median	Min	Max
University of California Los Angeles Loneliness Scale ^a^	41.0	10.1	39.5	21	69
absence or low sense of loneliness (<28.0)	*n* = 11 or 9.2%				
moderate sense (28.0–43.0)	*n* = 63 or 52.5%				
high sense (>43.0)	*n* = 46 or 38.3%				
Blood glucose, mg/dL	104.4	25.3	100.0	46	258
100+ or medication	*n* = 68 or 56.7%				
Total cholesterol, mg/dL	188.5	37.9	186.5	115	278
>200 or medication	*n* = 70 or 58.3%				
Triglycerides, mg/dL	110.3	58.6	97.0	39	319
>150 or medication	*n* = 46 or 38.3%				
HDL-C, mg/dL	59.5	18.4	56.0	26	144
<40/50 or medication	*n* = 24 or 20.0%				
LDL-C, mg/dL	108.0	29.9	107.4	47	186
>160 or medication	*n* = 40 or 33.3%				
LDL-C: HDL-C index	1.966	0.744	1.838	0.403	4.545
>2.517	*n* = 31 or 25.8%				
Fibrosis-4 index	1.193	0.489	1.165	0.350	3.090
1.45+	*n* = 28 or 23.3%				
Creatinine Clearance (CrCl), mL/min/1.73 m^2^	77.4	21.8	74.0	31	167
<90	*n* = 90 or 75.0%				

^a^ Score ranges between 20 and 80 as higher score indicates greater sense of subjective loneliness.

**Table 4:** Hierarchical multiple logistic regression analysis of higher levels of loneliness in relation to basic characteristics, health habits, blood glucose, serum lipids, Fibrosis-4 index, and Creatinine Clearance (CrCl) of 120 patients–attendees within primary care setting of current study.

	University of California Los Angeles Loneliness Scale (UCLA) (High Sense of Loneliness vs. Moderate/Low Sense)		
	1st Model	2nd Model	3rd Model
	Odds Ratio (95%CI) [*p*-Value]		
Gender (female vs. male)	0.77 (0.32, 1.85) [0.553]	0.63 (0.20, 2.02) [0.440]	0.87 (0.22, 3.42) [0.845]
Age (for every 5 year change)	1.09 (0.82, 1.46) [0.538]	1.21 (0.85, 1.71) [0.301]	1.31 (0.85, 2.03) [0.227]
Family status (married vs. unmarried, divorced, widow)	**0.39** (0.17, 0.91) [0.030]	0.38 (0.14, 1.07) [0.066]	0.34 (0.10, 1.10) [0.071]
Children (for each additional child)	0.61 (0.34, 1.08) [0.088]	0.66 (0.34, 1.27) [0.214]	0.67 (0.31, 1.44) [0.302]
Education (for each level of increase)	0.94 (0.71, 1.26) [0.695]	1.06 (0.75, 1.50) [0.731]	1.12 (0.75, 1.68) [0.578]
Occupation (unemployed, retired vs. employed)	0.50 (0.19, 1.31) [0.156]	0.36 (0.11, 1.17) [0.090]	0.39 (0.11, 1.35) [0.136]
Body Mass Index (overweight vs. normal)		0.51 (0.19, 1.39) [0.186]	0.47 (0.14, 1.60) [0.229]
Smoking (yes vs. no)		1.35 (0.48, 3.77) [0.568]	2.44 (0.77, 7.77) [0.130]
Alcohol consumption, drinks per week (yes vs. no)		2.63 (0.85, 8.20) [0.095]	**5.55** (1.42, 21.63) [0.014]
Individual sport/activity during the last year (yes vs. no)		**0.29** (0.10, 0.84) [0.023]	**0.28** (0.09, 0.91) [0.034]
Recent dramatic event in family during the last year (yes vs. no)		1.94 (0.73, 5.17) [0.185]	2.58 (0.83, 8.02) [0.101]
Routine contacts or meetings with friends during the last 6 months (for each additional person)		**0.82** (0.73, 0.93) [0.001]	**0.76** (0.66, 0.88) [<0.001]
Mental disorder (yes vs. no)		1.11 (0.42, 2.93) [0.826]	1.41 (0.47, 4.22) [0.540]
Blood glucose (100+ vs. <100 mg/dL)			1.04 (0.33, 3.26) [0.942]
Total cholesterol (>200 vs. ≤200 mg/dL)			0.45 (0.13, 1.58) [0.213]
Triglycerides (>150 vs. ≤150 mg/dL)			**0.20** (0.05, 0.83) [0.026]
LDL-C: HDL-C index (>2.517 vs. ≤2.517)			**4.50** (1.12, 18.13) [0.035]
Fibrosis-4 index (≥1.45 vs. <1.45)			1.19 (0.32, 4.38) [0.791]
Creatinine Clearance (<90 vs. ≥90)			0.83 (0.21, 3.30) [0.787]
R^2^ Negelkerke	0.146	0.380	0.507

Bold indicates the odds with *p*-value < 0.05.

## Data Availability

Datasets are available from the first author upon reasonable request.
